# Nanocodelivery of 5-Fluorouracil and Curcumin by RGD-Decorated Nanoliposomes Achieves Synergistic Chemotherapy for Breast Cancer

**DOI:** 10.1049/nbt2/4959295

**Published:** 2024-11-25

**Authors:** Reza Mahmoudi, Somayeh Mohammadi, Rouzbeh Mahmoudi, Mohamad Hassan Fouani, Maryam Tajali Ardakani, Amin Hadi, Mohsen Nikseresht, Mehrzad Jafari Barmak, Farzad Karimpour, Hassan Bardania

**Affiliations:** ^1^Cellular and Molecular Research Center, Yasuj University of Medical Sciences, Yasuj, Iran; ^2^Student Research Committee, Yasuj University of Medical Sciences, Yasuj, Iran; ^3^Department of Nanobiotechnology, Faculty of Biological Sciences, Tarbiat Modares University, Tehran, Iran; ^4^Social Determinants of Health Research Center, Yasuj University of Medical Science, Yasuj, Iran; ^5^Medicinal Plants Research Center, Yasuj University of Medical Sciences, Yasuj, Iran

**Keywords:** 5-fluorouracil, breast cancer, curcumin, drug delivery, liposome, RGD ligand

## Abstract

In the present study, arginine–glycine–aspartic acid peptide (RGD) surface functionalized liposomes (Lips) were formulated for the concomitant targeted delivery of two antineoplastic drugs, namely curcumin (Cur) and 5-fluorouracil (5FU) to breast cancer cells. The Lips' measured size values where 50–100 nm by transmission electron microscopy (TEM) and 169 ± 10.2 nm by dynamic light scattering (DLS), which fall within the desired range required for drug delivery purposes. In this study, we assessed the antineoplastic effects of various liposomal formulations for the codelivery of Cur and 5FU to MCF-7 breast cancer cells. We evaluated two liposomal formulations (Lip–Cur–5FU) and (Lip–Cur–5FU–RGD). The treatment of MCF-7 cells with 32 µg/mL of Cur exhibited a significant (*p* < 0.0001) drop in cell viability among the three formulations, namely Cur and 5Fu in the free form (Lip–Cur–5FU) and liposomal form (Lip–Cur–5FU–RGD); the least viability rate (9.91% ± 1.65%) corresponded to the RGD functionalized concomitantly Cur and 5Fu loaded Lips (Lip–Cur–5FU–RGD) formulation. On the other hand, liposomal Cur increased the rate of early apoptotic cell by 4.88% without altering the rate of late apoptotic cells. Furthermore, the concomitant treatment of MCF-7 cells with Cur and 5FU enhanced the overall apoptosis rate, where Cur–5FU in the RGD functionalized-liposomal form induced the highest (16.8%) apoptosis rate, while other Cur–5FU formulations, free and nonfunctionalized liposomal form, induced lower apoptosis rates (10.4% and 10.9%, respectively). Collectively our results demonstrated that the implementation of RGD-functionalized Lips for the concomitant delivery of Cur and 5FU enhanced their therapeutic efficacy against this breast cancer model.

## 1. Introduction

Breast cancer deemed a major global health concern, an estimate of 2.26 million cases was reported in 2020, is thought to be the most prominent cancer globally and leading cause of cancer death among females [1]. The most appropriate treatment method is prescribed based on cancer stage, the age of the patient, the risks, and benefits of each treatment method. Some of the methods of breast cancer treatment are surgery where the tumor is removed along with some of the surrounding tissues; radiation therapy where high-energy rays are implemented to kill cancer cells; chemotherapy which employs the administration of synthetic drugs administered intravenously or orally; doxorubicin, epirubicin, paclitaxel, 5-fluorouracil (5FU) and capecitabine are among chemotherapeutics prescribed for breast cancer treatment [2].

The broad-spectrum antineoplastic drug termed 5FU suppresses the action of the enzyme thymidylate synthase, which prevents deoxyribonucleic acid (DNA) synthesis and causes cytotoxicity and cell death [3]. However, short half-life due to its hydrophilic nature and rapid metabolism, wide distribution, in addition to various side effects undermines its medical applicability [4, 5].

On the other hand, curcumin (Cur), a polyphenol obtained from the rhizome of *Curcuma longa*, has been well recognized for its antineoplastic [6], anti-inflammatory [7], antimicrobial [8], and neuroprotective effects [9]. However, Cur's medicinal effectiveness is hampered by its poor solubility in water owing to its lipophilic nature, quick metabolism, high sensitivity to degradation, rapid clearance, and restricted absorption [10]. In addition, it was shown that Cur can improve the anticancer efficacy and decrease the side effects of chemical drugs such as 5FU [11].

Liposomes (Lips), polymeric nanoparticles, and inorganic nanoparticles are some examples of nanoparticle drug delivery systems (NDDSs) that have been widely employed to improve bioavailability and deliver therapeutic compounds site-specifically. Lips are small spherical vesicles synthesized using cholesterol and noncytotoxic phospholipids. Lips are promising NDDs owing to their biodegradability, biocompatibility, reduced toxicity, its amphiphilic nature which renders them capable of delivering both hydrophilic and hydrophobic molecules, and ease of surface modification [12, 13]. Lips' specificity and targeting efficiency could be further improved through surface functionalization using a variety of targeting molecules such as antibodies, peptides, aptamers, and hormones. The tripeptide arginine–glycine–aspartic acid (RGD) exhibits high affinity towards *α*v*β*3 cell surface receptor, which has elevated expression levels in several forms of cancer [12].

Several studies have been conducted demonstrating the capacity of Lips in enhancing the efficacy of antineoplastic drugs. For instance, Jiang, Shen, and Xu [14] simultaneously loaded Cur and paclitaxel into nano-Lips, and its effect on A549 human lung carcinoma cells xenograft mouse models was compared to unmodified nano-Lips counterpart. The results showed that the Cur–paclitaxel–RGD drugs had a greater antitumor effect in vivo compared to the unmodified Lip–Cur–paclitaxel drug [14]. In another study, Handali et al. [15] used folic acid (FA) to enhance Lips loaded with fluorouracil drug efficacy. In vitro and in vivo assessments against HT-29 and Henrietta Lacks (HeLa) cells demonstrated the FA functionalized Lips containing 5FU showed higher cytotoxicity compared to free fluorouracil and bare liposomal fluorouracil; FA functionalized Lips induced necrosis in HT-29 cells, while in HeLa cells FA functionalized Lips increased the apoptotic pathway by activating cytochrome c and caspase activity [15]. Similarly, Li et al. [16] investigated the effect of RGD-functionalized nano-Lips loaded with equimolar amounts of vinorelbine, tetrandrine, and daunorubicin on C6 glioma cell xenograft in rats. The test results demonstrated that RGD functionalized nano-Lips loaded with both vinorelbine and tetrandrine were more effective than both RGD functionalized vinorelbine loaded nano-Lips. However, RGD functionalized nano-Lips loaded with both vinorelbine and tetrandrine exhibited a similar efficacy to the unmodified counterpart containing both tetrandrine and vinorelbine and higher efficacy in comparison to vinorelbine Lips. Additionally, the results showed that the use of daunorubicin and tetrandrine with RGD Lips had a higher efficacy compared to the combination of tetrandrine with daunorubicin loaded bair Lips and had a similar efficacy to RGD functionalized vinorelbine loaded Lips [16]. Furthermore, in a study reported by Katarotia et al. [17], Lips containing 5FU were used to reduce cardiac toxicity and increase half-life; Ascorbyl-6-palmitate was also used to synergize with fluorouracil to combat cancer cells. Assessment against breast cancer cell line (MCF-7) demonstrated that the prepared formulation had greater anti-tumor activity. The cardiac toxicity assessment showed that stabilized Lips had minimal cardiac toxicity compared to the free drug [17]. In a study by Mahmoudi et al. [12], the active ingredient of turmeric (Cur) was encapsulated in RGD-modified Lips (RGD–Lip–Cur). The cytotoxic effect on the MCF-7 breast cancer cell line was evaluated using 3-[4,5-dimethylthiazol-2-yl]-2,5 diphenyl tetrazolium bromide (MTT), flow cytometry, and caspase assays. The MTT assay showed that RGD–Lip–Cur had a significant cytotoxic effect on MCF-7 cells at different Cur concentrations. The apoptosis assay showed that RGD–Lip–Cur significantly induced apoptosis in MCF-7 cells. Additionally, the caspase assay showed that RGD–Lip–Cur significantly activated caspases compared to Lip–Cur and Cur without nano-Lips. As a result, RGD–Lip–Cur was recognized as a new carrier with high cytotoxicity on the MCF-7 breast cancer cell line [12].

In the present study, RGD surface functionalized Lips were formulated aiming at the concomitant site-specific delivery of 5FU and Cur to breast cancer cells. It is expected that entrapment of 5FU and Cur in RGD functionalized Lips will enhance both efficacy and bioavailability and subsequently improve therapeutic results.

## 2. Materials and Methods

### 2.1. Materials

Chloroform, acetonitrile, methanol, and deionized water all high-performance liquid chromatography (HPLC) grade along with 5FU were purchased from Merck (Germany). Cur was supplied from Bio Basic (Canada). 1,2-Distearoyl-sn-glycero-3-phosphocholine (DSPC) was supplied from Avanti Polar Lipid (USA). MTT and cholesterol were purchased from Sigma (Germany). Penicillin-Streptomycin (Pen-Strep), RPMI1640 culture medium, and fetal bovine serum (FBS) were supplied from Gibco (USA). Dipalmitoyl-GRGDSPA was synthesized, and characterization data were previously disclosed in our paper [18].

### 2.2. Preparation of Lipid Formulations

Thin-film hydration method previously reported was used to prepare various liposomal formulations [19]. Briefly, using a round-bottomed flask Cur, cholesterol, dipalmitoyl-GRGDSPA and DSPC were mixed in chloroform at 1 : 1:1 : 7 molar ratios. Subsequently, in order to evaporate chloroform and obtain a thin lipid film, the flask was connected to a rotor evaporator (55°C, 150 rpm) under vacuum. The dried lipid film was hydrated with Tris-buffer containing 5FU at a final concentration of 2 mg/mL for passive 5FU loading.

### 2.3. Lip Characterization

The average size distribution of as-generated Lips was determined using dynamic light scattering (DLS) spectroscopy on a Malvern Zetasizer 3000 (Malvern Instruments, UK); measurements were performed at room temperature and repeated in triplicates.

Lips were also imaged using transmission electron microscopy (TEM). In brief, 5 µL of distilled water-diluted Lips were dried on the surface of a formvar-carbon-coated copper grid (Electron Microscopy Sciences, Hatfield, PA, USA). Phosphotungstic acid solution (2%) was put on the grid and allowed to dry for negative staining; TEM pictures were captured using a JEM-1010 microscope (JEOL, Tokyo, Japan).

### 2.4. Encapsulation Efficiency (EE)

HPLC was used to assess EE%; for chromatographic separation, a Eurospher 100-5 C18 column (200 4.6 mm, 5 m, KNAUER, Germany) was used. For measuring Cur concentration, the mobile phase was a 30 : 70 (*v*/*v*) combination of acetonitrile and water (including 5% acetic acid), with a constant flow rate of 1.15 mL/min; detection was conducted at 420 nm. On the other hand, the mobile phase for measuring 5FU concentration was a 50 : 50% (*v*/*v*) combination of acetonitrile and water (containing 0.1% orthophosphoric acid), with a constant 1 mL/min flow rate; detection was conducted at 260 nm.

### 2.5. Assessment of Antineoplastic Efficacy by Means of MTT Assay

MTT assay was used to assess the antineoplastic effects of Cur and/or 5FU loaded Lips against MCF-7 cells, as previously described [12]. Cells were treated with various formulations: free (Cur) and liposomal Cur (Lip–Cur) (4, 8, 16, and 32 µg/mL of Cur); free (5FU) and liposomal 5FU (Lip–5FU) (62.5, 125, 250, 500, and 1000 µg/mL of 5FU); RGD modified Lips loaded with both Cur and 5FU (Lip–Cur–5FU–RGD), and unmodified Lips loaded with both Cur and 5FU (Lip–Cur–5FU); cells receiving no treatment were considered control group, for 24 h. For combinatory delivery, a constant concentration of 5FU (250 µg/mL) was used with different concentrations of Cur. The absorbance of the samples was recorded at 570 nm using an ELX800 ultraviolet (UV) universal microplate reader (Bio-Tek Instruments Inc., Vermont, USA). Untreated control was normalized to 100%, whereas treated groups were stated as the percentage of the control group; each assay was repeated in triplicate. The ratio of the viable cells was determined based on the following formula:

  $$\begin{array}{c} \text{Cell viability }\left(\%\right)=100\times A_{s}/A_{c}, \end{array}$$

where *A*_*s*_ and *A*_*c*_ depict the absorbance in the sample and the control group, respectively. Results are expressed as the mean ± standard deviation (SD).

### 2.6. Apoptosis Assay

Apoptosis rate was quantified by fluorescein isothiocyanate (FITC) tagged Annexin V and propidium Iodide (PI) fluorescent staining (BioLegend, San Diego, USA), as previously described [20]. The cells were treated by 250 µg/mL of 5FU and 16 µg/mL of Cur for different groups. Rate of viable cells, early apoptotic cells, late apoptotic cells, and necrotic cells was assessed using the automated multicolor flow cytometry system, BD FACS (fluorescence activated cell sorting) Calibur Flow Cytometer (BD Biosciences, USA); results analysis was performed using Flowing 2.5.1 software.

### 2.7. Statistical Analysis

GraphPad Prism software package (GraphPad Software Inc., USA) was used to collect data, which were expressed as average ± SD. Experiments were conducted in triplicates and verified through repetition; analysis of variance (ANOVA) was used for the ANOVA, and then Tukey correction was applied.

## 3. Results and Discussion

### 3.1. Characterization of Lip–Cur–5FU–RGD Lips

**Figure 1:**
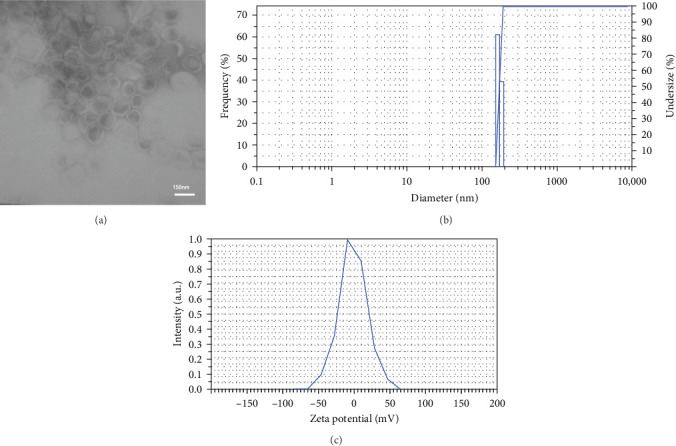
TEM image of Lip–Cur–5FU–RGD formulation (A); size (B), and zeta potential (C) assessment by DLS analysis. 5FU, 5-fluorouracil; Cur, curcumin; DLS, dynamic light scattering; Lip, liposome; RGD, arginine–glycine–aspartic acid peptide; TEM, transmission electron microscopy.

Size is one of the physical criteria that affect Lips' circulation half-life as well as the amount of Lips' loading capacity, where 50–200 nm size of Lips is desired for drug delivery application [21]. Additionally, nanocarriers can extend the half-life of antineoplastic drugs through the enhanced permeability and retention (EPR) effect, causing a greater concentration of chemotherapeutic drugs in the tumor area [22]. In this study, Figure 1 exhibits Lips' measured size values where 50–100 nm by TEM microscopy (Figure 1A) and 169 ± 10.2 nm by DLS (Figure 1B), which fall within the desired range required for drug delivery purposes.

### 3.2. EE

Determination of EE% of a drug into Lips is important in the context of evaluation of the effectiveness of the encapsulation method [23]. EE% of Cur into Lips was measured to be ~99%, which is higher than previously reported formulations. On the other hand, EE% of 5FU loaded into Lips was measured to be 55%, where it is lower from some reports 62% [23] and 59% [24], and higher than others 31% [25].

### 3.3. In Vitro Cytotoxic and Apoptotic Effects of Various Cur−5FU Formulations

**Figure 2:**
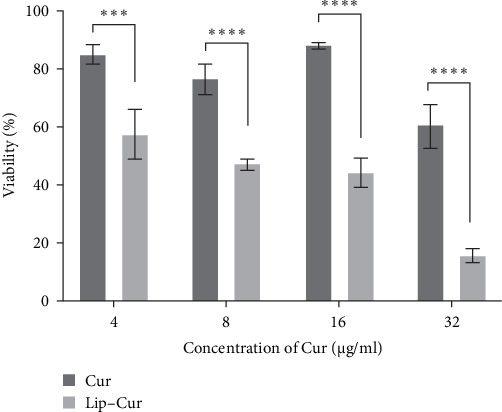
The effect of curcumin encapsulation into liposome on its cytotoxicity against MCF-7 cell line. Cur, curcumin; Lip, liposome (*⁣*^*∗∗∗*^*p* < 0.001; *⁣*^*∗∗∗∗*^*p* < 0.0001).

**Figure 3:**
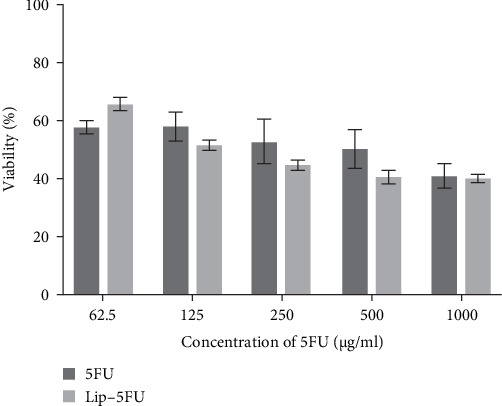
The effect of 5FU encapsulation into liposome on its cytotoxicity against MCF-7 cell line. 5FU, 5-fluorouracil; Lip, liposome.

**Figure 4:**
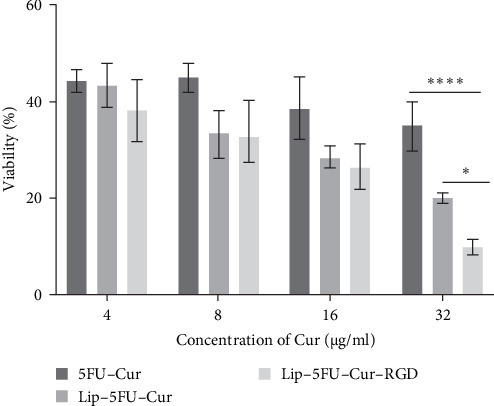
The effect of coencapsulated curcumin and 5FU into RGD-functionalized liposome on its cytotoxicity against MCF-7 cell line; a constant concentration of 5FU (250 µg/mL) was used with different concentrations of Cur. 5FU, 5-fluorouracil; Cur, curcumin; Lip, liposome; RGD, arginine–glycine–aspartic acid peptide (*⁣*^*∗*^*p* < 0.05; *⁣*^*∗∗∗∗*^*p* < 0.0001).

Several reports have established Cur's potential to increase the antineoplastic efficacy of chemotherapeutic agents against several types of malignancies including breast cancer [26, 27]; the coadministration of Cur has been found to boost antineoplastic effect of chemotherapeutic agents including 5FU [28]. In this study, the antineoplastic effects of liposomal formulations loaded with both Cur and 5FU were assessed against MCF-7 breast cancer cells. As depicted in Figure 2, upon 24 h of treatment of MCF-7 breast cancer cells, liposomal Cur (Lip–Cur) significantly (*p* < 0.001) dropped cell proliferation in comparison to the free form (Cur), at all tested concentrations, in a concentration-dependent manner; no significant toxicity was detected for Cur in the free form, except for 32 µg/mL concentration. This finding is consistent with earlier studies that showed that the cytotoxic effects of Cur were noticeably enhanced when they were enclosed in NDDs such Lips or nanoparticles based. On the other hand, both liposomal (Lip–5FU) and free form (5FU) induce toxicity in a concentration-dependent manner; however, no significant variation was detected between the two 5FU formulations. Furthermore, the coadministration of Cur and 5FU in the free form caused a drop in cell viability, with a mild variation in cell toxicity upon increment in their concentration (Figure 3). To further assess the efficacy of 5FU, we evaluated two liposomal formulations (Lip–Cur–5FU) and (Lip–Cur–5FU–RGD). As depicted in Figure 4, only the treatment of MCF-7 cells with 32 µg/mL of Cur exhibited a significant (*p* < 0.0001) drop in cell viability among the three formulations, namely Cur and 5Fu in the free form (Lip–Cur–5FU) and liposomal form (Lip–Cur–5FU–RGD); the least viability rate (9.91% ± 1.65%) corresponded to the RGD functionalized concomitantly Cur and 5Fu loaded Lips (Lip–Cur–5FU–RGD) formulation.

**Figure 5:**
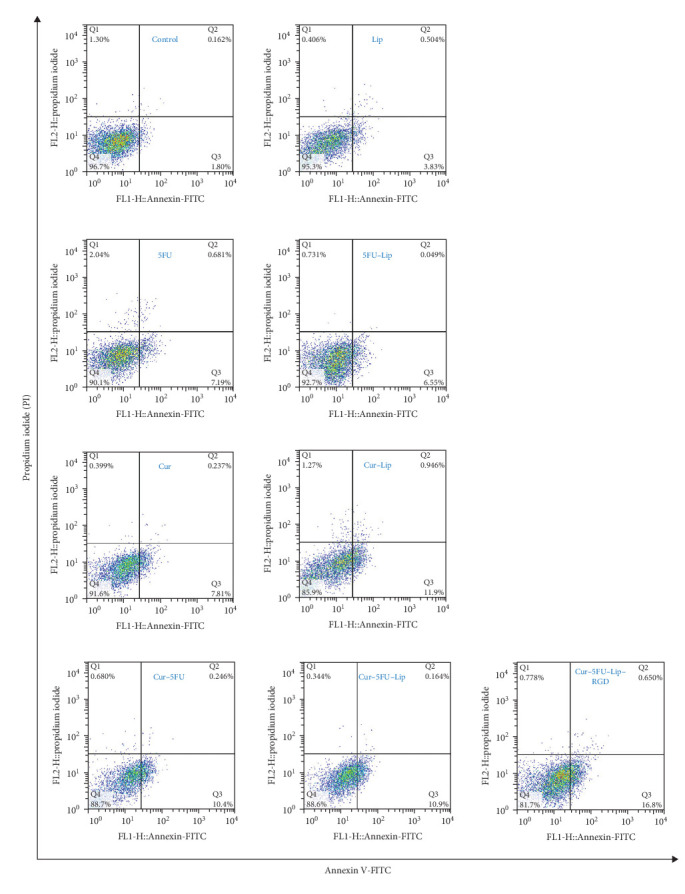
The apoptotic effect of coencapsulated curcumin and 5FU into RGD-functionalized liposome against MCF-7 cell line. 5FU, 5-fluorouracil; Cur, curcumin; Lip, liposome; RGD, arginine–glycine–aspartic acid peptide.

Though through different mechanisms of action, both Cur and 5FU induce apoptosis in MCF-7 cells [29, 30]. To this aim, the apoptotic effect of various Cur–5FU formulations was assessed against MCF-7 cells using flow cytometry through Annexin V-FITC/PI staining. In this procedure, FITC tagged Annexin V binds phosphatidylserine (PS) which translocates from the internal layer of the plasma membrane to the external one, marking early apoptosis. Furthermore, PI discriminates between dead cells and viable cells, through fluorescent DNA binding after the passive permeation across the plasma membrane of dead cells; PI cannot permeate across viable cells' plasma membrane [12]. As depicted in the dot–plot graphs of the flow cytometry assay (Figure 5), the percentage of viable cells (Annexin V^−^/PI^−^) is shown in the lower left quadrant (Q4), percentage of the early-phase apoptotic cells (Annexin V^+^/PI^−^) ^−^) are shown in the lower right quadrant (Q3), percentage of late-phase apoptotic cells (Annexin V^+^/PI^+^) are shown in the upper right quadrant (Q2) and percentage of necrotic cells (Annexin V^−^/PI^+^) is shown in the upper left quadrant (Q1); total apoptosis is determined by summing early apoptosis (Q3) and late apoptosis (Q2) quadrants. All assessed Cur–5FU formulations exhibited insignificant necrosis rates in MCF-7 cells (Figure 5). The proportion of early apoptotic cells exposed to 5FU in both free (7.19%) and liposomal (6.55%) form did not differ significantly; a similar situation was observed in late apoptotic cells exposed to free 5FU (0.681%) and liposomal 5FU (0.049%) (Figure 5). On the other hand, when compared to its free form, liposomal Cur increased the rate of early apoptotic cell by 4.09% without altering the rate of late apoptotic cells (Figure 5). Furthermore, the concomitant treatment of MCF-7 cells with Cur and 5FU enhanced the overall apoptosis rate, where Cur–5FU in the RGD-functionalized liposomal form induced the highest (16.8%) apoptosis rate, while other Cur–5FU formulations (Figure 5); free and nonfunctionalized liposomal form, induced lower apoptosis rates (10.4% and 10.9%, respectively) (Figure 5). These observations imply that the antineoplastic synergistic effect of liposomal Cur and 5FU, on MCF-7 breast cancer cells, is directly proportional to encapsulated Cur concentration. This finding is consistent with previously published studies describing the concentration-dependent modulatory effects of Cur when combined with 5FU or other antineoplastic drugs. According to Zhang et al. [31], uptake through *α*v*β*3 integrin receptor is governed by both ligand-specificity and saturation sensitivity, hence, it is possible that excess RGD peptide could block the *α*v*β*3 receptor uptake, resulting in decreased uptake of RGD modified Lips. This fact might interpret the ~10% difference in cytotoxicity between unfunctionalized (Lip–Cur–5FU) and RGD functionalized (Lip–Cur–5FU–RGD) formulation, and the necessity of the evaluation of (Lip–Cur–5FU–RGD) Lips with lower RGD modification degree.

## 4. Conclusion

Conventional cancer therapeutics suffer from several drawbacks, mainly side effects, low bioavailability; NDDSs have been implemented to enhance bioavailability and efficacy of the drugs. Passive and active approaches have been employed for targeted drug delivery, where active targeting uses different ligands to target specific receptors present on cancer cells' surface. Several reports exist on the enhancement of Lips' drug delivery of both hydrophobic and hydrophilic drugs to cancer cells upon functionalization with RGD peptide through targeting *α*v*β*3 receptor.

Collectively our results demonstrated that the implementation of RGD-functionalized Lips for the concomitant delivery of Cur and 5FU enhanced their therapeutic efficacy against this breast cancer model. To our knowledge, RGD functionalized liposomal formulation harboring both Cur and 5FU has never been evaluated in preclinical models for breast cancer.

## Data Availability

The datasets generated and/or analyzed during the current study are available from the corresponding author upon reasonable request.
